# Use of hormonal contraception and attempted suicide: a nested case-control study

**DOI:** 10.1192/j.eurpsy.2022.339

**Published:** 2022-09-01

**Authors:** E. Toffol, T. Partonen, A. Latvala, A. But, O. Heikinheimo, J. Haukka

**Affiliations:** 1 University of Helsinki, Department Of Public Health, Helsinki, Finland; 2 Finnish Institute for Health and Welfare, Department Of Public Health And Welfare, Helsinki, Finland; 3 University of Helsinki, Institute Of Criminology And Legal Policy, Helsinki, Finland; 4 University of Helsinki and Helsinki University Hospital, Department Of Obstetrics And Gynaecology, Helsinki, Finland

**Keywords:** hormonal contraception, nested-case control, women, attempted suicide

## Abstract

**Introduction:**

In Finland more than 40% of fertile aged women used some type of hormonal contraception (HC) in the period 2010-2013. A proportion of women using HC complains of side effects, including mood symptoms. The relationship between the use of HC and the risk of attempted suicide (AS) is still a matter of debate.

**Objectives:**

To assess the association of the use of HC with the risk of AS during 2017-2019.

**Methods:**

Data were retrieved from the Prescription Centre, Care Register of Health Care, Register of Primary Health Care Visits and Statistics Finland. A total of 587 823 women, aged 15-49 years, using and not using HC in 2017 were analysed in the initial incidence study. All incident AS cases during 2018-2019, and their 4:1 age-matched controls (1 174 346 person-years) were analysed in a nested case-control setting via conditional logistic regression models.

**Results:**

Altogether 818 AS cases occurred during the follow-up (incidence rate: 0.70/1000 person-years, 95% CI 0.65–0.75), with an IRR of HC vs. no-HC use of 0.73 (0.63–0.83). Current use (in the 180 days before the event) of estradiol- or ethinylestradiol-containing HC was associated with a lower risk of AS (0.53, 0.33–0.87; 0.49, 0.37–0.64, respectively) compared to non-use of HC. After controlling for covariates (marital and socioeconomic status, education level, use of psychotropic medications), only current use of HC containing ethinylestradiol remained significant (0.39, 0.23–0.65).

**Conclusions:**

A lower risk of AS is associated with the use of HC, and specifically of ethinylestradiol-containing HC.

**Disclosure:**

No significant relationships.

